# Protective Efficacy of a *Plasmodium vivax* Circumsporozoite Protein-Based Vaccine in *Aotus nancymaae *Is Associated with Antibodies to the Repeat Region

**DOI:** 10.1371/journal.pntd.0003268

**Published:** 2014-10-16

**Authors:** Anjali Yadava, Cysha E. Hall, JoAnn S. Sullivan, Douglas Nace, Tyrone Williams, William E. Collins, Christian F. Ockenhouse, John W. Barnwell

**Affiliations:** 1 Malaria Vaccine Branch, Military Malaria Research Program, Walter Reed Army Institute of Research, Silver Spring, Maryland, United States of America; 2 Malaria Branch, Division of Parasitic Diseases and Malaria, Centers for Disease Control and Prevention, Atlanta, Georgia, United States of America; Federal University of São Paulo, Brazil

## Abstract

We have previously reported that Vivax Malaria Protein 001 (VMP001), a vaccine candidate based on the circumsporozoite protein of *Plasmodium vivax*, is immunogenic in mice and rhesus monkeys in the presence of various adjuvants. In the present study, we evaluated the immunogenicity and efficacy of VMP001 formulated with a TLR9 agonist in a water-in-oil emulsion. Following immunization, the vaccine efficacy was assessed by challenging *Aotus nancymaae* monkeys with *P. vivax* sporozoites. Monkeys from both the low- and high-dose vaccine groups generated strong humoral immune responses to the vaccine (peak median titers of 291,622), and its subunits (peak median titers to the N-term, central repeat and C-term regions of 22,188; 66,120 and 179,947, respectively). 66.7% of vaccinated monkeys demonstrated sterile protection following challenge. Protection was associated with antibodies directed against the central repeat region. The protected monkeys had a median anti-repeat titer of 97,841 compared to 14,822 in the non-protected monkeys. This is the first report demonstrating *P. vivax* CSP vaccine-induced protection of *Aotus* monkeys challenged with *P. vivax* sporozoites.

## Author Summary

*Plasmodium vivax* is responsible for causing malaria in large parts of the globe, including regions with temperate climates not suited for the transmission of other *Plasmodium* species. In addition, *P. vivax* has the propensity to form dormant forms, known as hypnozoites, that can remain latent for weeks to months and reactive periodically to cause recurrent infections. Prevention of *P. vivax* malaria, more than any other form, will require a vaccine-based intervention due to limitations in treatment options. To this end, we tested the efficacy in non-human primates, of a vaccine based on circumsporozoite protein, a preerythrocytic stage antigen, of *P. vivax. Aotus* monkeys were immunized with clinical-grade antigen, combined with two immunomodulators, and then challenged with *P. vivax* sporozoites. Following challenge 66.7% of monkeys were protected. Analysis of serum samples indicated that protection was associated with antibodies to the central repeat region of the molecule, and that protection was lost upon waning of these antibodies. This is the first report demonstrating that active immunization with a recombinant protein can lead to complete protection in monkeys following sporozoite challenge, while also demonstrating a protective associate. Our data can help serve as a benchmark for down-selection of future vaccine formulations for *P. vivax*.

## Introduction

The range of *Plasmodium vivax* transmission spans 95 countries putting 2.86 billion people at risk for this malaria parasite [1] and causes an estimated 132–391 million infections each year [2]. In addition to its widespread distribution, *P. vivax* also has the propensity to form dormant hypnozoites in the liver, which reactivate periodically and result in recurring relapse infections. Currently, the only treatment for these intrahepatic hypnozoites is the 8-aminoquinoline, primaquine (PQ), which is contraindicated in a variable proportion of populations due to a risk of hemolysis in individuals with G6PD deficiency [3] or during pregnancy. More recently, Bennett and colleagues reported an association between decreased activity of the CYP2D6 isoenzyme and reduced metabolism of PQ resulting in treatment failure [4]. This further reduces the pool of individuals who may be treated with PQ, reinforcing the need to develop a vaccine to prevent *P. vivax* malaria. However, resources for vivax research remain limited, with only 5% of malaria funds specifically directed toward *P. vivax* between 2007 and 2011 (PATH Malaria R&D Report, 2013). In addition, funding initiatives such as the U.S. government's President's Malaria Initiative (PMI) have strictly limited assistance, mostly to select countries in Africa, leaving little room for funding vivax malaria control or research [5].

Due to the unpredictability of hypnozoite reactivations that cause relapse infections, an intervention based on a preerythroctyic stage antigen is even more imperative for *P. vivax* to prevent primary infection and subsequent relapse infections. The circumsporozoite protein (CSP) is the major protein present on the surface of sporozoites and is involved in hepatocyte binding and invasion and as such is the lead vaccine candidate for malaria. Presence of CSP on hypnozoites [6] makes it an attractive target against both the sporozoite, and intrahepatic parasites. We have designed and produced a vaccine based on the CSP of *P. vivax* and demonstrated its antigenicity and immunogenicity [7] [8].

Rodents serve as a platform for the initial screening of malaria vaccine candidates. However, non-human primates, being closer to humans, are more suitable models to assess vaccines. A limited number of studies have been performed to analyze immunogenicity, and even fewer to assess the efficacy, of candidate vaccines for *P. vivax* in non-human primates. In the late 1980s and early 1990s studies were performed with recombinant *P. vivax* CS proteins produced in yeast [9] and *E. coli* (WRAIR-SKB), which gave little to no protection in immunized *Saimiri* monkeys [10]. Subsequently, multiple antigen constructs were used to develop epitope-based vaccines using the vivax repeat motif. Protection was observed in *Saimiri* monkeys [11] [12], but the lack of a control group makes it difficult to conclusively interpret the data in these studies. An *Aotus* monkey model was used to assess immunogenicity of CS multiple antigen peptides (MAP) and long synthetic peptides (LSP) and cells from immunized monkeys were used to delineate T-cell epitopes on the protein [13] [14]. No challenge studies were performed with the MAP or LSP immunized monkeys to correlate the immunogenicity to protection.

In this study, we evaluated the immunogenicity and protective efficacy of VMP001, a soluble *P. vivax* CSP-based vaccine, co-formulated with a TLR-9 agonist and a water-in-oil emulsion. Immunized *A. nancymaae* monkeys demonstrated high antibody titers and high efficacy following challenge with *P. vivax* sporozoites. Vaccine efficacy was shown to be associated with antibodies to the repeat region of *P. vivax* CSP. To our knowledge, this is the first report of an efficacy study of a recombinant CSP-based vaccine in *Aotus nancymaae* monkeys, and the first to conclusively correlate this protection to antibodies generated against the repeat region of the CSP following immunization.

## Materials and Methods

### Immunogens and antigens

The vaccine antigen, designated Vivax Malaria Protein 001 (VMP001), is based on the *P. vivax* CSP and has been described previously [7], [8]. Briefly, the gene encoding a chimeric central repeat region containing the nonameric repeat motifs from both VK210 (nine copies) and VK247 (one copy) parasites and flanked by the N- and C-terminal regions of the *P. vivax* CSP was cloned into a plasmid under a T5 promoter, expressed in *Escherichia coli*, and purified under cGMP. The purified vaccine antigen did not have detectable endotoxin, both by an in vitro assay, as well as by in vivo pyrogenicity testing in rabbits [8].

*P. falciparum* Liver Stage Antigen 1 (*Pf*LSA-1), kindly provided by Dr. David Lanar [15], was used as an unrelated malaria antigen for immunizing the control group of animals. Recombinant proteins encoding the N-terminal and C-terminal regions of *P. vivax* CSP were produced in *E. coli* for use as plate antigens in humoral assays. A 36-mer peptide representing four copies of the classical Type 1 repeat motif was used to detect antibodies to this repeat region.

### Ethics statement

The research using non-human primates (*Aotus nancymaae* and *Aotus lemuirinus grisiemembra*) reported in this manuscript was conducted under animal protocol number 1588BARMONB and was approved by the Institutional Animal Care and Use Committee (IACUC) of the Centers for Disease Control and Prevention. The non-human primate (NHP) research was conducted in compliance with the Animal Welfare Act and other U. S. federal government statutes and regulations relating to animals and research involving animals All NHP research adhered to the principles stated in the Guide for the Care and Use of Laboratory Animals, National Research Council. The New World monkeys used in this study were either captive bred or imported for research from Peru under a program administered through PAHO and NIH for research purposes and were socially pair-housed in appropriately spacious stainless steel cages in a BSL2-facility at the CDC vivarium facilities in Atlanta, Georgia, USA under controlled conditions of humidity, temperature, and light (12-hour light/12-hour dark cycles). This facility is fully accredited by the Association for the Assessment and Accreditation of Laboratory Animal Care International and has an approved OLAW Assurance #A4365-01. The animals were provided with commercial high protein food biscuits and supplemented with appropriate fresh vegetables, fruits and treats. Drinking water was provided ad libitum. Enrichment was provided in the form of hollow pieces of large tubing to simulate tree trunk cavities, mirrors, food puzzles and perches. Animals were monitored daily for health and discomfort. A large experienced staff is available including full time veterinarians and a pathologist. All steps were taken to ameliorate the welfare and to avoid any suffering of the animals in accordance with the recommendations of the Weatherall report for the use of nonhuman primates in research. Major surgeries (splenectomy) were performed with full anesthesia in aseptic surgical suites by an experienced surgical veterinarian. Monkeys were not euthanized during this research. Vivax malaria infections were treated with standard antimalarial drugs; quinine, chloroquine and primaquine as noted previously.

### Immunizations and bleeds

Male and female monkeys were randomly assigned to three groups of eight animals each. Two test groups received either 15 µg or 50 µg of VMP001 per immunization. A third group of monkeys, which served as controls, was immunized with 50 µg *P. falciparum* LSA-1. All antigens were formulated in Montanide ISA 720 (Seppic, NJ) (antigen to adjuvant ratio of 30∶70) plus 200 µg of CpG 10104 (Coley Pharma) per immunization. Animals were immunized intramuscularly in the thigh muscles, alternating legs for each immunization, with approximately 300 µl of each vaccine formulation on day 0, 28 and 114. Monkeys were bled two weeks prior to immunization and at two-week intervals following vaccinations for the collection of serum. Serum was separated by centrifugation, aliquoted and frozen at −70°C until use.

### Challenge

Sporozoites were isolated from salivary glands of *Anopheles dirus* that were infected with the Brazil VII strain of *P. vivax* after feeding on an infected *A. lemurinus grisemembra* monkey (Barnwell et al. manuscript in preparation). The Brazil VII strain of *P. vivax* carries a type I (VK210) *csp* gene (J. Barnwell, unpublished data). Each monkey was challenged intravenously with 10,000 sporozoites six weeks after the third immunization. Two to three days following sporozoite challenge all the monkeys were splenectomized under general anesthesia and sterile operating conditions. Splenectomies were performed to allow for the establishment of a synchronous blood stage infection resulting in a more uniform, and shorter, prepatent period.

Blood stage parasitemia was monitored in thick and thin Giemsa stained blood smears starting approximately 14 days post-challenge. Monkeys that demonstrated patent blood stage infection were treated with 50 mg/kg of quinine for seven days. Parasitemia was monitored on a daily basis until the conclusion of second phase of the study (described below).

Kaplan-Meier survival curves were generated for each group individually, as well as for pooled (low- and high-dose) vaccinated groups, and plotted as percentage of uninfected monkeys over time. Survival curve comparisons were performed using Log-rank (Mantel-Cox) test. Overall vaccine efficacy (VE) was calculated as 1-R×100, where R = I_v_/I_c_ i.e. the ratio of Incidence of malaria in vaccinated group (I_v_) to the Incidence of malaria in the control group (I_c_).

At eight weeks post primary challenge all surviving monkeys were rechallenged intravenoulsy with a higher dose of 15,000 *P. vivax* Brazil VII sporozoites. Monkeys that demonstrated patent blood infection were treated with quinine as above.

### ELISAs

Sera from monkeys were tested for the presence of CSP antibodies following immunization. Immulon 2HB plates (Dynatech, VA) were coated with 0.4 µg/ml VMP001, N- and C-terminal regions of *P. vivax* CSP or 1 µg/ml of Type 1 peptide. Sera from a selected time point were also screened against *PfLSA1* to determine if the control group generated antibodies against the transgene, and to ensure that there was no cross reactivity between the control and vaccine groups. Plates were blocked, incubated with serially diluted serum, followed by 1∶6000 dilution of a custom prepared goat anti-*Aotus* IgG (CDC, Atlanta) labeled with horseradish peroxidase (HRP). The reaction was developed with ABTS and read after 60 min at *A*_414_. ELISA titers are defined as the serum dilution that gives an optical density (OD_414_) of 1.0.

### Statistical analysis

GraphPad Prism version 5.0 was used for statistical analysis. Tests used for each analysis are mentioned along with the corresponding data.

## Results

### Immunogenicity of VMP001 vaccine in *Aotus* – Analysis of anti-VMP001 antibodies

**Figure 1:**
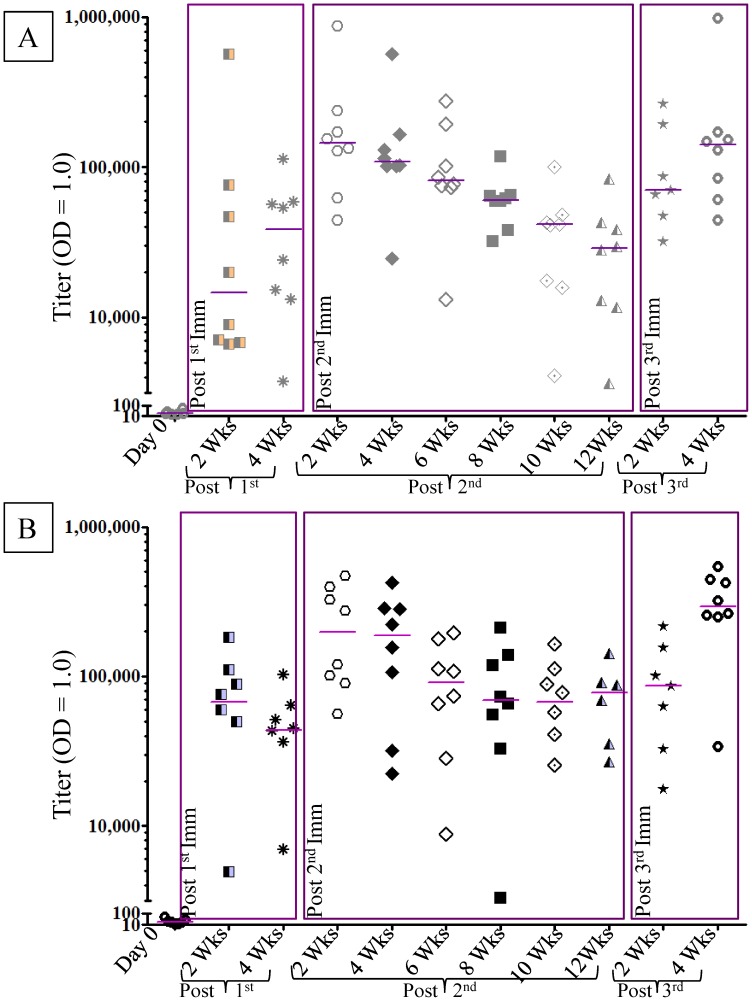
*Aotus nancymaae* immunized with VMP001 generated antigen-specific antibodies that were boosted following each immunization. Groups of 8 monkeys were immunized three times with either 15 µg (panel A) or 50 µg (panel B) of VMP001 formulated with 200 µg CpG 10104 and Montanide ISA 720. Sera, drawn at two week intervals, were tested for presence of antibodies to VMP001. Antibody titers waned over time, but were boosted with each immunization. Titers are defined as serum dilution that gives an OD_414_ of 1.0. Each symbol represents an individual monkey. Some time points have less than eight data points due to the unavailibility of serum sample at that time point. Horizontal bars represent the median titer for each group at the given time point.

Monkeys were immunized with either 15 µg (low dose) or 50 µg (high dose) of VMP001formulated with Montanide ISA 720 in combination with 200 µg of CpG 10104, a TLR-9 agonist. This study was designed with an ultimate goal of assessing the efficacy of the VMP001 vaccine formulation. Adjuvants are known to induce innate immune responses which can affect the outcome of a challenge. Therefore, in order to rule out the role of non-specific immune responses we immunized a group of monkeys with *P. falciparum* LSA-1 that served as an unrelated malaria antigen, formulated in adjuvant. Monkeys were immunized three times, with a one month interval between the 1^st^ and 2^nd^ immunization, and a three month interval between the 2^nd^ and 3^rd^ immunizations. Animals were monitored following each immunization and no reactogenicity or adverse events were observed. None of the control monkeys that received *Pf*LSA-1 generated any antibodies against VMP001 following immunization, however, robust anti-*Pf*LSA1responses were detected in this group, while none of the VMP001 immunized sera showed any anti-*Pf*LSA1 reactivity (data not shown). Anti-VMP001 antibodies were detected in all monkeys immunized with VMP001 two weeks following the 1^st^ immunization (Figure 1). The median titers at 2 weeks post 1^st^ immunization were 14,581 and 67,695 for the low and high dose groups, respectively. These titers were boosted significantly at 2 weeks post 2^nd^ immunization for both the low dose (144,302; *p* = .02) and high dose (198,024; *p* = .02) groups. Antibody titers declined over time and the titers were significantly lower in both immunized groups at 12 weeks after the 2^nd^ immunization compared to the peak titers obtained two weeks after the 2^nd^ immunization (*p* = .0006 and .015 for the low and high dose groups, respectively). Titers showed a boost following the 3^rd^ immunization and peaked 4 weeks post 3^rd^ immunization. There was a significant difference between the titers at 12 weeks post 2^nd^ immunization (28,940) and 4 weeks post 3^rd^ immunization (140,367; *p* = 0.006) for the low dose group and the high dose group (78,163 at 12 weeks post 2^nd^ immunization and 291,622 at 4 weeks post-3^rd^ immunization; *p* = .005). The peak titers post the 2^nd^ and 3^rd^ immunizations were not significantly different (Figure 1).

**Figure 2:**
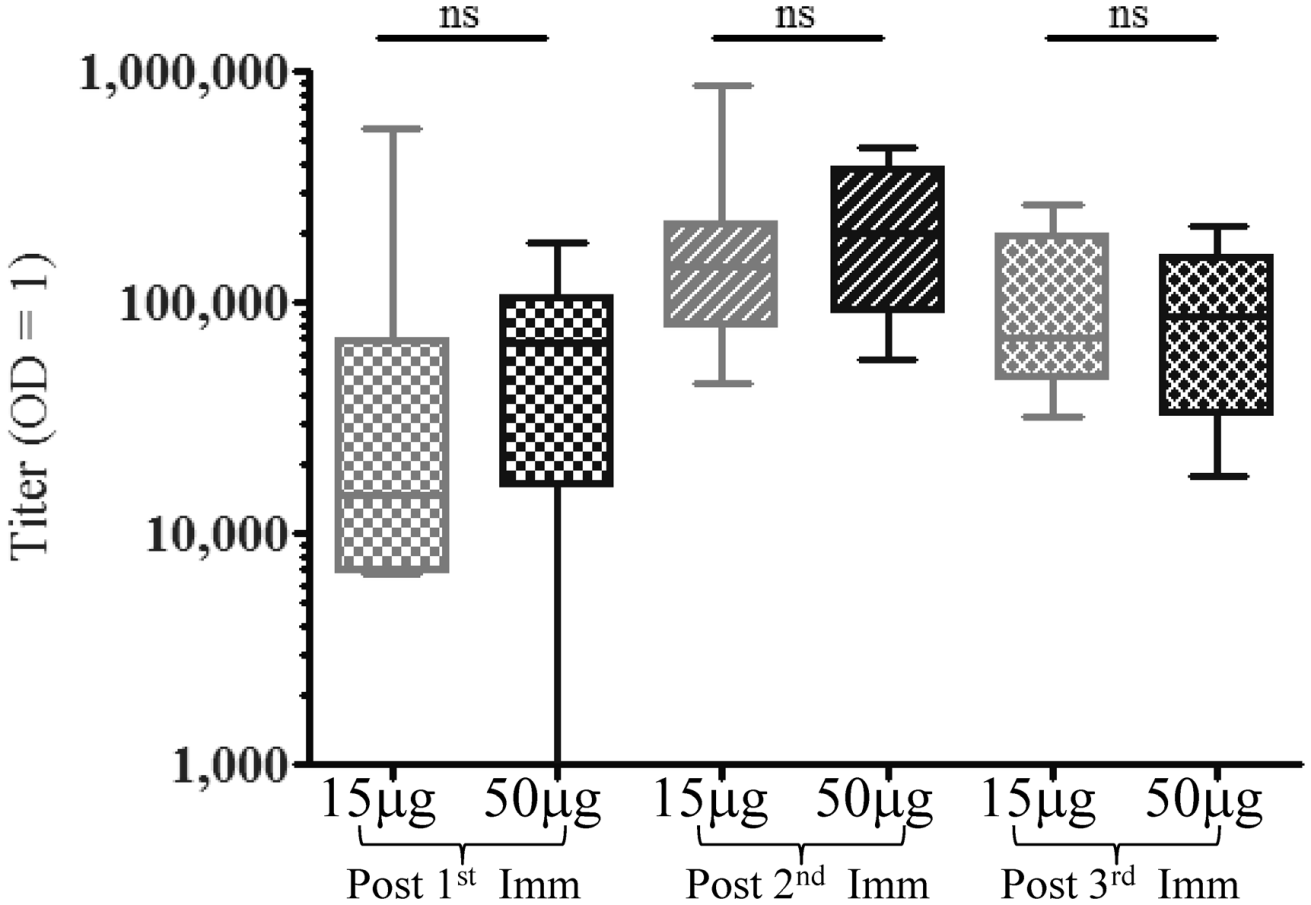
Onset and magnitude of antibody response to VMP001 is similar in both the low (15 µg) and high (50 µg) dose groups. In the presence of a strong adjuvant formulation the differences in anti-VMP001 titers between the two immunized groups were not stastically significant (ns = *p*>0.05; 2-tailed Mann Whitney U test) at any timepoint. Data is represented as Box (25–75 percentile) and Whisker (minimum and maximum) plot with horizontal line at the median value.

The antibody titers for both the low and high dose groups were similar at 2 weeks post each immunization (Figure 2). The median titers ranged from a low of 14,581 at 2weeks post 1^st^ immunization for the low dose group, to a high of 198,024 for the high dose group at 2 weeks post 2^nd^ immunization. There were no statistical differences in antibody titers between the groups at each time point indicating that a strong adjuvant overcomes antigen dose effects.

### Efficacy of VMP001 following intravenous challenge with *P. vivax* Brazil VII parasites

**Figure 3:**
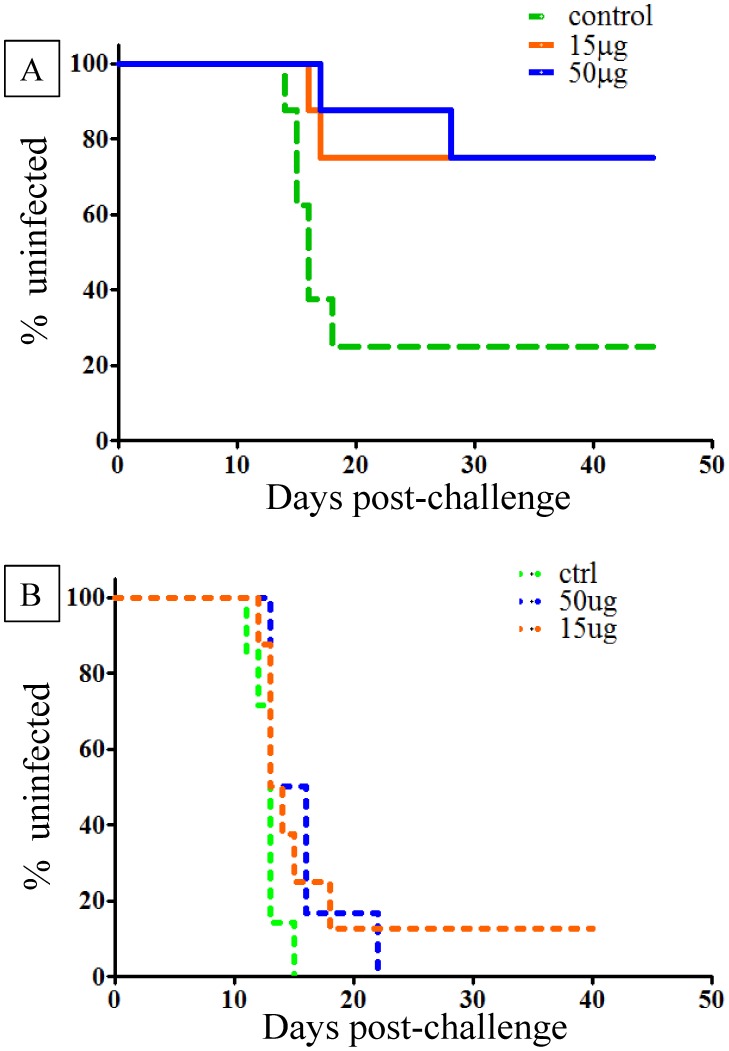
Protective efficacy of VMP001 in *Aotus* monkeys challenged intravenously with *P. vivax* Brazil VII strain post primary and rechallenge. Monkeys were immunized with either 15 µg or 50 µg of VMP001. Control monkeys were immunized with 50 µg of *P. falciparum* LSA-1 (*Pf*LSA-1). All antigens were formulated with Montanide ISA 720 and CpG 10104. Kaplan-Meier survival curves represent percentage of uninfected monkeys over time. Vaccine Efficacy was calculated as a ratio between the vaccinated and control groups (see results). **3A**. Six weeks following the third immunization, all monkeys were challenged intravenously with 10,000 *P. vivax* Brazil VII parasites. **3B**. Eight weeks after the primary challenge, monkeys were rechallenged with 15,000 *P. vivax* Brazil VII parasites.

Following intravenous inoculation of sporozoites, six of the eight (75%) *Pf*LSA-1 control monkeys became parasitemic, with a tight pre-patent period ranging from day 14 to 18 post-challenge (Figure 3A). In fact, five of the six infected monkeys had demonstrated patent infection by day 16. The remaining monkey became positive on day 18. Six of the eight monkeys in each of the two vaccinated groups did not demonstrate patent infection. In both the low and high dose groups 25% monkeys in each group (2/8) became parasitemic with a prepatent period (day 17 and 28 for the low dose group; day 16 and 17 for the high dose group) that was delayed in one animal compared to the controls. The median prepatent period for the control group was 15.5 days, 22.5 days for the low dose group and 16.5 days for the high dose group. Total vaccine efficacy, calculated by comparing the ratio of infected monkeys in the vaccinated vs. the control group, for each group was 66.7%; and the *p* values, based on the Mantel-Cox Log-rank test, for the time to parasitemia were .02 and .03 for the low and high dose groups respectively.

Following treatment, all surviving monkeys were rechallenged intravenously eight weeks post primary challenge with a higher dose of 15,000 *P. vivax* Brazil VII sporozoites. This allowed for an approximately two-week interval between the clearance of any primary infections before the rechallenge. Seven of seven control monkeys became parasitemic between days 11 and 15 (Median day 13) (Figure 3B). Six of six rechallenged monkeys in the high dose group became parasitemic between days 12 and 22 (Median day 14.5) and seven of eight of the low dose group became parasitemic between day 12 and 18 (Median day 13.5). One monkey in the low dose group did not become parasitemic during the 33 day post-rechallenge observation period.

### Induction of anti-VMP001 antibodies following challenge with *P. vivax* sporozoites

**Figure 4:**
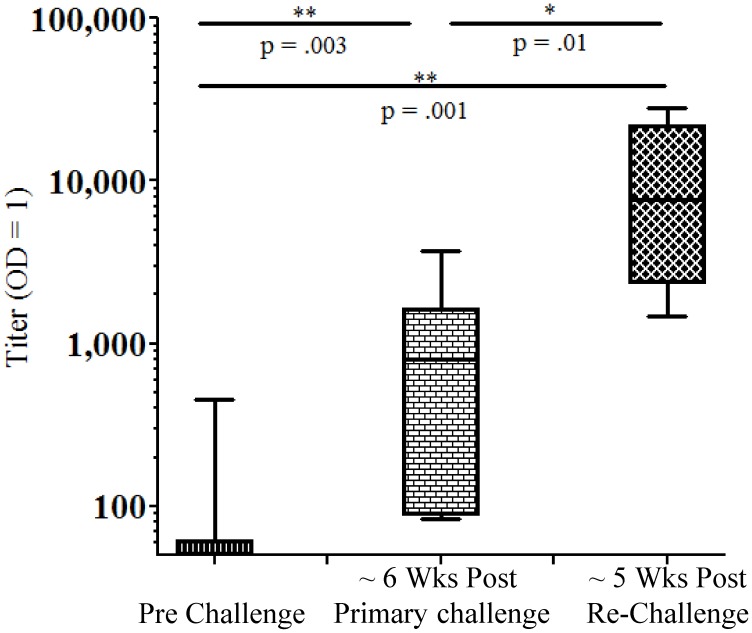
Infection with *P. vivax* Brazil VII sporozoites induces antibodies to VMP001. Control monkeys immunized with *Pf*LSA-1 did not generate antibodies against *P. vivax* (denoted as “pre challenge” in the figure). Following challenge with *P. vivax* sporozoites, the control monkeys developed anti-*P. vivax* CSP antibodies, as assessed by ELISA reactivity to VMP001, that were significantly boosted following rechallenge (2-tailed Mann-Whitney U test). Data is represented as Box (25–75 percentile) and Whisker (minimum and maximum) plot with horizontal line at the median value.

As expected, the control animals, immunized with an unrelated malaria antigen, *Pf*LSA-1, did not induce antibodies to VMP001 (Figure 4) after the 3^rd^ immunization. However, following primary challenge, these animals generated antibodies to CSP, as demonstrated by the presence of antibodies to VMP001 (Median titer: 797). The titers were boosted approximately 10-fold following rechallenge to a median titer of 7,670. The differences between the prechallenge and post-challenge anti-*Pv*CSP (VMP001) titers in the *Pf*LSA-1 group were statistically significant (Figure 4). Since the prechallenge anti-VMP001 titers in the vaccinated monkeys were already high, the differences in titers post-challenge were not significant.

### Immunogenicity of VMP001-antibody fine-specificity analysis

Serum from immunized monkeys was further analyzed to assess the breadth of antibody responses generated. At 4 weeks post 3^rd^ immunization (2 weeks pre-challenge) all monkeys demonstrated presence of antibodies to the N-, Central Repeat as well as the C-terminal region of *P. vivax* CSP. As with VMP001 (Median titers of 140,367 and 291,622 for the low and high dose groups respectively) there was no significant difference between the antibody titers against the N-term (Median 7,277 vs. 22,188), Type 1 repeat peptide (Median 66,120 vs. 25,297) and C-term regions (Median 77,111 vs. 179,947).

### Association between anti-Type 1 antibody levels and vaccine efficacy

**Figure 5:**
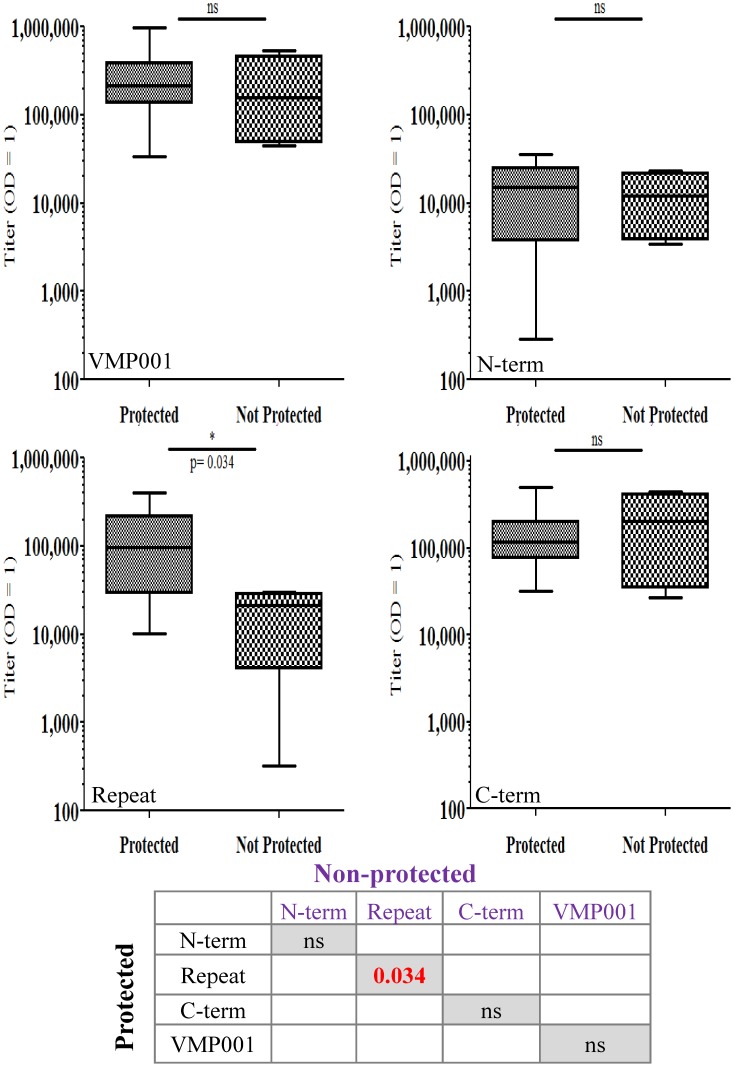
Protected monkeys from both the low- and high dose groups had significantly higher anti-repeat antibodies compared to the non-protected monkeys. Pre-primary challenge sera from immunized monkeys from the low (15 µg) and high (50 µg) dose groups were pooled from the protected (n = 12) and non-protected (n = 4) groups. Antibody reactivity between the protected and non-protected groups were similar for VMP001, N- and C-terminal regions. However, anti-repeat antibodies were significantly different between the protected and non-protected groups (*p* = 0.034, 2-tailed Mann Whitney U test). Data is represented as Box (25–75 percentile) and Whisker (minimum and maximum) plot with horizontal line at the median value. Data also represented in a tabular form to demonstrate correlation bewteen protected and non-protected groups.

The data was reanalyzed by pooling the protected (n = 12) and non-protected (n = 4) monkeys from both the high and low dose groups (Figure 5). There were no statistical differences between the VMP001 (Median 214,659 vs. 156,247), N-term (Median 14,979 vs. 11,952) and C-term (Median 116,291 vs. 202,126) titers. The titers to the Type 1 repeat peptide were significantly different between the protected (Median 97,841) and non-protected (Median 21,517) groups (*p* = 0.03; 2-tailed Mann-Whitney U test), indicating that high anti-repeat antibodies may be a correlate of protection. This is in accordance with data from *P. falciparum* vaccine studies where anti-repeat antibodies have been shown to be the strongest correlate of protection in humans [16].

**Figure 6:**
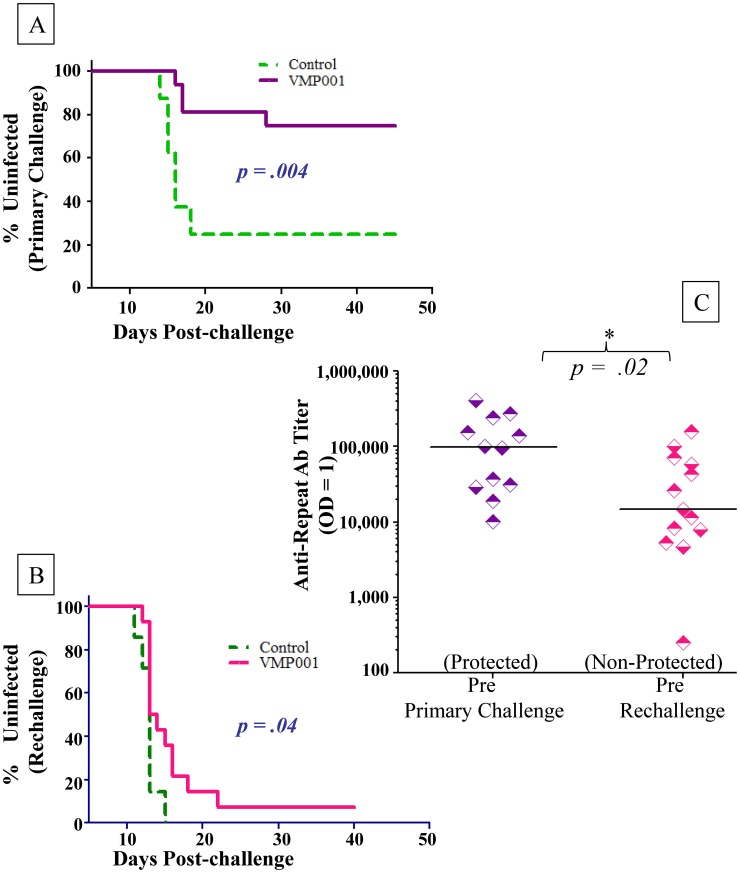
Loss of protective efficacy following rechallenge could be attributed to a drop in anti-repeat antibody titers. Kaplan-Meier survival curves were generated by pooling data from the low (15 µg) and high (50 µg) dose groups (from Figure 3), and the combined VMP001 immunized group was compared to the control group. **6A**. Following primary challenge, 12 of 16 immunized monkeys and 2 of 8 control monkeys remained uninfected, resulting in a calculated vaccine efficay of 66.7% (p = 0.004). **6B**. Following rechallenge, one immunized monkey remained uninfected and several others had a delay compared to the controls (*p* = .04). **6C**. Sera drawn two weeks prior to the first challenge from protected monkeys (n = 12) had significantly higher anti-repeat antibody titers compared to sera drawn pre-rechallenge from the non-protected monkeys (n = 13); p = 0.02 (2-taled Mann Whitney U-test). Titers indicate serum dilution that gives an OD_414_ of 1.0. Each dot represents an individual monkey. Horizontal bar represents the median titer for each group.

As stated earlier, all but one of the vaccinated monkeys that were protected following primary challenge (Figure 6A) lost their protection upon rechallenge (Figure 6B). Nevertheless, when data from the low- and high-dose groups were combined following rechallenge and compared to the controls, there was still a significant difference between the immunized and control groups (*p* = 0.036) (Figure 6B). In comparison, following primary challenge the combined vaccinated groups had a *p*-value of 0.004 compared to the controls (Figure 6A).

We therefore compared the antibody titers in the prechallenge sera from the monkeys that were protected prior to the primary challenge to the titers in sera from those that were not protected following rechallenge. Sera were drawn two weeks before the primary-, or re-challenge. Here too, the only significant differences were found between the anti-repeat antibodies. As shown in Figure 6C the median anti-Type 1 repeat antibody titer in the protected monkeys was 97,841 at the time of primary challenge compared to 14,822 (*p* = 0.02) in the monkeys that were not protected following rechallenge. Thus, the loss of protection in the previously protected monkeys could be attributed to waning of the anti-repeat antibodies. Antibodies to VMP001 also declined (Median 214,569 vs. 97, 296) but the decrease was not statistically significant. There was no association between antibody titers and time to patency in the monkeys that became parasitemic.

## Discussion

Due to financial, logistical, and scientific challenges, efforts to develop vaccines for *P. vivax* have been disproportionately slower compared to that for *P. falciparum*. In addition to the limited number of preclinical studies in non-human primates that we describe above, a recent review by Reyes-Sandoval and Bachman [17] highlights the meager clinical portfolio of *P. vivax* vaccines and the current status of vivax research and funding. In sharp contrast to *P. falciparum*, so far, only one efficacy study has been conducted in humans (clinical trials registration number NCT01157897, Bennett et. al. in preparation). There is a need to test products to down-select candidate(s) that can be advanced to the clinic.

Towards this end, we produced a recombinant CSP-based vaccine and assessed its immunogenicity in rhesus monkeys using two different TLR4 agonists as adjuvants. In a study using a synthetic TLR4 agonist, glucopyranosyl lipid A (GLA), formulated in oil (GLA-SE), we demonstrated the safety and immunogenicity of VMP001. The vaccine induced potent humoral responses directed to the different regions of the molecule, and a cellular response predominantly directed to the C-terminal region of the protein [18]. In another study, rhesus monkeys were immunized with soluble recombinant protein (VMP001), as well as its particulate counterpart CSV-S,S which is a fusion between VMP001 and the hepatitis B surface antigen to compare the immunogenicity of the two formulations in AS01, an adjuvant approved for human use [19]. Both the soluble and particulate vaccines induced strong humoral and cellular immune responses, but with some qualitative and quantitative differences between the two formulations.

The present study was undertaken to test the immunogenicity, as well as efficacy, of VMP001 in *Aotus* monkeys. We immunized monkeys with two different doses of antigen formulated in Montanide ISA 720 and CpG 10104. Montanide ISA 720 is a water-in-oil emulsion constituted of a nonmineral metabolizable oil and a mannide monooleate surfactant [20]. CpG 10104 is a B-class 24-mer synthetic oligonucleotide with a fully phosphorothioate backbone containing 5 CpG motifs (Coley Pharma). These unmethylated CpG DNA motifs are recognized by mammalian TLR9 and are known to activate dendritic cells, macrophages and B cells [21]. Both Montanide ISA 720 and CpG have been used in clinical studies in humans with malaria antigens (www.clinicatrials.gov) and thus, have a possible path forward for the clinical evaluation of vaccine candidates. Therefore, we chose to evaluate our vaccine in the presence of these adjuvants to take advantage of their synergistic roles in inducing a functionally-relevant immune response. Due to the possible effect of the innate immune responses induced by this combination of adjuvants on the outcome of a parasite challenge, we included a control arm in which the monkeys were injected with the adjuvant formulated with an unrelated *P. falciparum* antigen that does not exist in *P. vivax*. The final vaccine efficacy was estimated by comparing it to the outcome of the control group. Due to the fact that 25% of the control monkeys did not become parasitemic, our calculated vaccine efficacy for both the low and high dose groups singly, and in combination, following the primary challenge was 66.7% with a total of 12 out of 16 immunized monkeys remaining free of blood stage parasites. Both the low- and high-dose groups induced robust immune responses to the whole recombinant protein (VMP001), as well as the N-term, repeat- and C-term regions of the protein. However, only anti-repeat antibodies showed a statistical association with protection. The twelve protected monkeys had statistically significant antibody response that was 4.5-fold higher compared to the response in the non-protected monkeys. In contrast, the protected and non-protected monkeys had differences of 1.7-fold, or less, in anti-VMP001, N- and C-term antibody titers. While similar comparisons with the various components (the entire immunogen, or the C-term region) have not been published for RTS,S the only subunit malaria vaccine to show consistent protection in humans, evaluation of anti-repeat antibodies has been shown to be a strong correlate of protection [16]. In a previous study, passive transfer of a mouse monoclonal antibody directed against the repeat region of the *P. vivax* CSP was shown to protect 4/6 monkeys [22]. However, immunization with *Pv*CSP recombinant proteins failed to induce protective anti-repeat antibodies [22] [10].

The strength of our protective correlate to the anti-repeat titers is further bolstered by the results of the rechallenge experiment. Since only one monkey stayed protected after the rechallenge, it was not possible to compare the protected vs. non-protected groups post-rechallenge. Therefore, we compared the anti-Type 1 repeat antibody titers in the monkeys that were protected post-primary challenge vs. those that were not protected post-rechallenge. The antibody titers showed a significant decline from a median of 97,841 pre-primary challenge to 14,822 pre-rechallenge. This 6.6-fold drop in titers was statistically significant (p = .02) and explains the loss of protection in the previously protected monkeys.

We would be remiss in not stating, as pointed out by one of the reviewers, that T cell responses were not evaluated in this study. In addition, this study was not designed to test effectiveness against in situ hypnozoites and it is possible that this vaccine may or may not be effective against established hypnozoites, and thus may need to be reformulated and associated with other antigens.

In summary, we demonstrate VMP001 vaccine-induced protection in the presence of strong immunomodulators, Montanide ISA 720 and a TLR9 agonist. The presence of one, or both, of these components may have caused a qualitative shift in the immune response to drive the production of high-titers of anti-repeat antibodies to a level that resulted in protection. The results of this study—using the nonhuman primate model of the *P. vivax* Brazil VII strain in *A. nancymaae*—can help serve as a benchmark for down-selection of adjuvant formulations in future studies with the current vaccine antigen, or while designing new antigens to serve as a vaccine.
